# Synthesis of graphene aerogels using cyclohexane and *n*-butanol as soft templates†

**DOI:** 10.1039/c9ra10988a

**Published:** 2020-04-08

**Authors:** Xin Zhang, Jie Su, Xueyuan Wang, Xiaolei Tong, Fanglian Yao, Caideng Yuan

**Affiliations:** Department of Polymer Science and Engineering, School of Chemical Engineering and Technology, Tianjin University Tianjin 300350 P. R. China cdyuan@tju.edu.cn; Tianjin University-UCGM Joint Research Center of Graphene Application, Tianjin University Tianjin 300350 P. R. China

## Abstract

Graphene aerogels (GAs) were synthesized *via* a one-step hydrothermal method. Generally, the pore shape and diameter of GAs are difficult to control or the preparation process is complicated, requiring a multi-step operation. Herein, a soft-template one-step hydrothermal synthesis process was proposed to produce GAs with controllable pore sizes. Cyclohexane and *n*-butanol were added to a graphene oxide suspension to form a uniform aqueous dispersion under emulsification by sodium lauryl sulfate. The reduction process may have occurred around the organic droplets during the hydrothermal reaction, and a large number of organic droplets became countless physical barriers inside the hydrogel. In the later freeze-drying and high-temperature calcination procedures, the droplets evaporated to form a rich pore structure. Compared to the conventional templating method, the organic template was volatilized during the drying process such that no additional process for removing the template was required. In addition, GAs prepared by the template method possessed lower density (2.66 mg cm^−3^) and better compression performance and, as an adsorbent material, absorbed organic matter and petroleum from wastewater more efficiently than GAs obtained by the traditional one-step hydrothermal method; *Q* for *n*-hexane reached 116, and *Q* for xylene reached 147; also, the GAs prepared by the soft template method can absorb all crude oil in water samples within 30 s.

## Abstract 2

Graphene aerogels (GAs) were synthesized *via* a one-step hydrothermal method.

## Graphical abstract

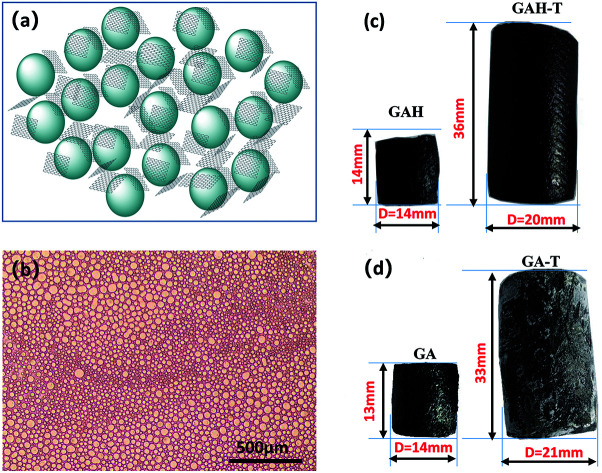

## 1. Introduction

Graphene has excellent electrical and mechanical properties and can be widely applied in supercapacitors,^1–3^ batteries,^1,3–5^ sewage treatment,^6–8^ photocatalysis,^9–11^ and sensors.^12^ However, graphene, an unstable two-dimensional (2D) material, can reduce its energy through irreversible agglomeration, such that it is difficult to exert its full performance in practical applications. Therefore, three-dimensional (3D) graphene aerogels (GAs) have attracted more attention. GA has an ultrahigh specific surface area,^2,12^ high electrical conductivity,^13^ very low density,^8,13,14^ abundant pore structure,^15–18^ and certain hydrophobic and lipophilic properties,^19^ and it not only effectively retains the properties of graphene but is also easy to assemble into parts for use in macroscopic materials.

In recent years, researchers have developed a variety of methods to assemble 2D materials into 3D forms in more efficient and applicable ways, including chemical reduction,^20^ chemical crosslinking,^21^ and polymerization.^22^ Graphene oxide (GO) contains many oxygen-containing functional groups and is often used as a precursor for the construction of graphene aerogels. GO can be uniformly dispersed in a solvent, such as water, using ultrasound. In a hydrothermal process^23^ using a solvent or a chemical reduction with a reducing agent,^24^ GO loses most of its oxygen-containing functional groups and is converted to reduced GO (rGO). Because of its low hydrophilicity, rGO cannot be stably dispersed in water and the resulting layers stack spontaneously to form a stable hydrogel under the action of π–π bonding interactions. However, due to the special 2D structure of GO, with a very high aspect ratio, rGO is more inclined to stack *via* self-assembly rather than chaotic aggregation. During the stacking process, rGO sheets undergo a hydrophobic action that captures large amounts of solvent to form a graphene hydrogel (GAH). The density, pore size, and wall thickness of the GAH are randomly synthesized and cannot be precisely controlled and, thus, a template method has been proposed. There are two mature ideas for the synthesis of GAHs and/or GAs by the template method; one is a chemical vapor deposition (CVD) process and the other is a solution assembly method. The CVD method mainly deposits graphene directly onto commercially available foamed nickel^25^ or a special functional template.^26^ The process maximizes the excellent properties of graphene and allows for custom shapes through using a stencil, but the CVD process is relatively expensive and can only produce small quantities in the laboratory, with, in most cases, the original stencil etched away. In contrast, self-assembled templates are relatively simple to use and GA can be produced in mass and, thus, are widely used.

An ice template process, called ice-segregation-induced self-assembly (ISISA),^27–29^ is considered to be the easiest method for constructing 3D graphene aerogels. In the process of synthesizing a GAH, when the system is about to reach the gel point, the precursor is immediately frozen in liquid nitrogen or in a refrigerator and ice crystals formed during the freezing process are used as templates. Before the gel point, the GO has been partially deoxidized into a hydrophobic material, such that the ice crystals divide the system into a porous structure. The GAH formed by the reheating reaction has a special pore structure, which can be controlled by the freezing temperature, freezing time, and freezing direction. At the same time, however, the pore size of the GA synthesized by the ISISA method is generally nonuniform, mainly due to the difference in temperature gradients at different locations. Porous solid monoliths have also been used by many researchers as templates for GA synthesis, such as some common polymeric porous materials,^30,31^ silica,^32^ and even everyday sponges. Different from the CVD method, these monoliths can accommodate large amounts of GO suspension. And GO is then reduced to rGO using an appropriate method and the template is etched by calcination or strong acid to form a 3D porous GA. The porous monolithic template can adjust the pore size and distribution very conveniently according to needs and the prepared GA has good mechanical strength, chemical properties, and thermal stability. However, the etching process is time consuming and environmentally unfriendly. Although no high temperature treatment is needed during the etching process, further high temperature reactions are required to obtain better electrical performance compared to the CVD method. Pickering emulsions are also a popular method for preparing GA materials. A Pickering emulsion utilizes solid particles^33–35^ as an emulsifier and has better stability than conventional emulsions. As an amphiphilic particle, GO can form a stable oil-in-water (O/W) emulsion. The monomer is polymerized as a foreign phase to form a skeleton and then the solvent is removed to obtain a polymer/graphene monolith. However, to obtain a pure GA, it is still necessary to etch away the already-formed polymer, thus limiting its application. Shi^36^ proposed a better and simpler method for controlling the pore structure. By dispersing a certain amount of *n*-hexane into the GO suspension, GO is reduced and assembles into a 3D structure in the hydrothermal reaction. Droplets of *n*-hexane do not disperse GO and, being incompatible with water, form a spherical physical barrier, such that the pore size of the formed GAH is affected by the oil droplet size, which can be controlled by adjusting the amounts of *n*-hexane and stirring, and thus the GA pore size can be predetermined. A further advantage is that the removal of *n*-hexane as a template does not require an additional step, with *n*-hexane being removed along with water during dialysis and freeze-drying. However, *n*-hexane droplets are easily separated from water without the aid of an emulsifier, resulting in an uneven pore diameter in the GA upper and lower positions, thus the method needs to be further improved.

In this study, unstable water/*n*-hexane systems were improved by introducing cyclohexane and *n*-butanol into GO suspensions and forming more stable aqueous cyclohexane–butanol/GO emulsions with the help of emulsifiers and dispersers. The prepared GO emulsions were then used to synthesize graphene hydrogels by a one-step hydrothermal method and the obtained hydrogels, named GAH-Ts, were then freeze-dried to form graphene aerogels, named GA-Ts. To obtain better graphene performance, residual oxygen atoms were removed from GA-Ts by calcination to form aerogels (FGAs) with improved graphene structures. By adjusting the ratio of aqueous to organic phases (cyclohexane and butanol), the pore structures of these hydrogels and aerogels, as well as the final FGA structure, were adjusted.

## 2. Experimental

### 2.1 Chemical materials

Flake graphite (325 mesh) was supplied by Unigram Carbon Graphene Materials Co., Ltd, (Hebei, China; UCGM). Concentrated sulfuric acid (H_2_SO_4_), cyclohexane, *n*-butanol, HCl, KMnO_4_, ethylenediamine (EDA, 99.5%), hydrogen peroxide (H_2_O_2_), phosphoric acid (H_3_PO_4_), and sodium dodecyl sulfate (SDS) were all analytical grade and purchased from Aladdin Chemistry Co. Ltd (Shanghai, China). All chemicals were used as received.

### 2.2 Preparation of graphene hydrogels and aerogels

**Scheme 1:**
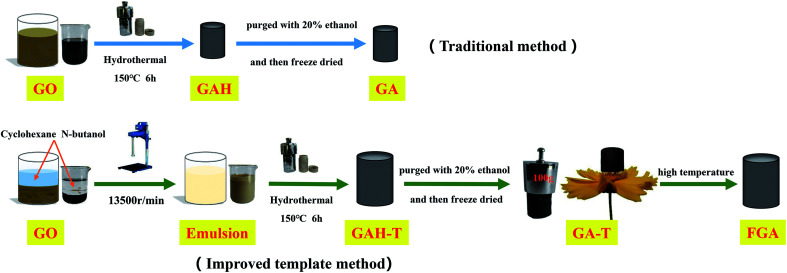
Schematic diagram of the formation process of graphene hydrogels and aerogels with the conventional hydrothermal method and improved template method.

GO was prepared by a modified Hummers' process and the detailed steps can be found in the ESI.† The basic experimental procedure for preparing graphene hydrogels and aerogels is shown in Scheme 1. In brief, 50 ml of GO suspension (6 mg ml^−1^) was mixed with cyclohexane (50 ml) and *n*-butanol (37.5 ml) in a 150 ml beaker and then SDS (0.9 g) as emulsifier and 250 μl of EDA as reducing agent were added to the mixture. The mixture was dispersed by subsequent treatment with a high-speed homogenizer at 13 500 rpm for 5 min to obtain an off-white uniform emulsion. After the emulsion was degassed under vacuum, 10 g of the emulsion was fed into a hydrothermal kettle with a Teflon lining. Graphene hydrogels (GAH-Ts) were then obtained by keeping the hydrothermal kettle in a 150 °C muffle furnace for 6 h. GAH-T was carefully purged with 20% ethanol solution and freeze-dried for 48 h to form absolutely dry aerogels (GA-Ts). The product was further calcined at 600 °C for 5 h under N_2_ protection in a high temperature tube furnace such that GA-Ts were completely reduced to form the porous aerogels (FAGs).

For comparison, graphene hydrogels and aerogels were also prepared *via* the conventional hydrothermal method, in which only GO suspension (6 mg ml^−1^) and EDA (reducing agent) were used in the hydrothermal reaction without a soft template (cyclohexane and butanol). These obtained hydrogels and aerogels were termed GAH and GA, respectively.

### 2.3 Characterization

The morphology of the graphene hydrogels (or aerogels) was characterized by using scanning electron microscopy (SEM; Zeiss Merlin Compact, Carl Zeiss AG, Oberkochen, Germany). The chemical functional groups were characterized by using Fourier transform-infrared spectroscopy (FT-IR; Vertex 70, Bruker Corp., Billerica, MA, USA). The crystal structure was characterized by using X-ray diffraction (X'Pert PRO MPD, Malvern Panalytical B.V., Almelo, The Netherlands), operating with Cu-K_α_ radiation (*λ* = 0.15418 nm) at a scan rate (2*θ*) of 5° min^−1^. Raman spectra of the samples were recorded using a laser Raman spectrometer (DXR, Thermo Fisher Scientific Inc., Waltham, MA, USA) with an excitation wavelength of 532 nm. Surface element composition analysis was performed by using Thermo ESCALAB Model 250XI X-ray photoelectron spectroscopy (XPS, Thermo Fisher Scientific Inc.), in which the binding energy was calibrated to the C-1s peak (284.8 eV) with a reference.^37^ Thermal gravimetric analysis (TGA) was performed using a thermal analyser (SDT Q600, TA Instruments, New Castle, DE, USA) under a nitrogen flow of 20 ml min^−1^, at a heating rate of 10 °C min^−1^, and in a range from 25 to 800 °C. The surface roughness and morphology of GO were measured by using atomic force microscopy (AFM, Dimension Icon, Bruker Corp.) in tapping mode and with 256 × 256-pixel resolution. Samples for AFM imaging were prepared by spin-coating the GO dispersion in water (1 × 10^−5^ mg ml^−1^) onto a freshly cleaved mica surface (200 rpm and 60 s) and allowing it to dry in air.

### 2.4 Adsorption capacity test of graphene aerogels

The solvent/oil adsorption capacity was measured according to the ASTM F726-99: Standard Test Method for Sorbent Performance of Adsorbents. Here, *n*-hexane and xylene were selected as representatives of hydrocarbons and aromatic hydrocarbons, respectively, and a water/crude oil mixture as a model for spilled-oil wastewater.

Graphene aerogel samples (GA or GA-T) were weighed (*w*_1_) and then placed in a 150 ml beaker, to which 100 ml of organic solvent (*n*-hexane or xylene) was added. The adsorbent was removed after 60 s of immersion, gently wiped with filter paper, and then immediately weighed (*w*_2_).

For evaluation of adsorbent performance in spilled oil adsorption, 5 g of crude oil was mixed with 95 g of distilled water in a beaker to obtain the simulated petroleum-contaminated wastewater. The subsequent test method was the same as that for the organic solvent above. The adsorption capacity (*Q*) was calculated using the weight of the aerogels before (*w*_1_) and after adsorption (*w*_2_) as
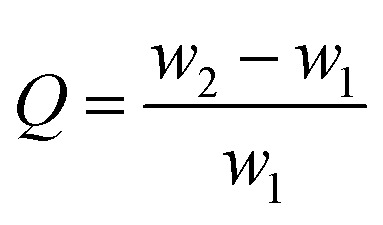


## 3. Results and discussion

### 3.1 Morphology of graphene hydrogels and aerogels

Scheme 1 illustrates the synthetic process for the graphene aerogel prepared by the conventional method (GAH and GA) and the improved soft-template method (GAH-T and GA-T). GA was prepared by a hydrothermal reaction without any templates, while, in contrast, GA-T was obtained by a hydrothermal reaction with cyclohexane and *n*-butanol as soft-templates. GA-T exhibited high strength and did not collapse under a 100 g weight (typically its own weight was 30 mg) and the density was very low (<10 mg cm^−3^), such that it could stand on a flower easily (Scheme 1).

**Fig. 1:**
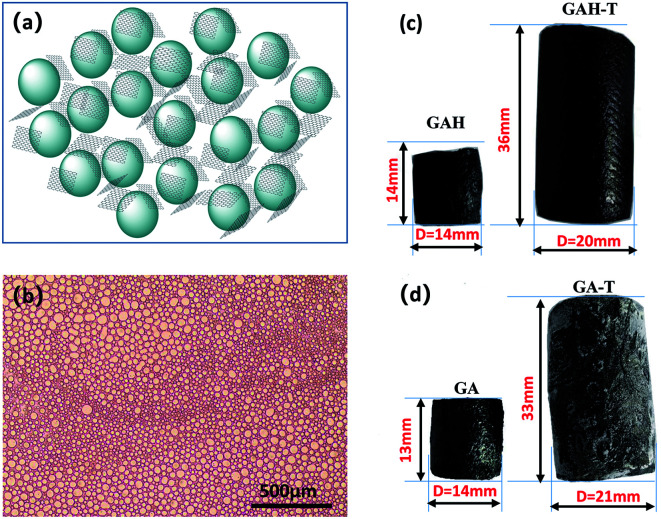
Schematic diagram of an oil phase droplet-induced porous structure (a). Optical micrograph of the template emulsion (b). Physical photographs of graphene hydrogels prepared by the conventional hydrothermal method (GAH) and the improved soft-template method (GAH-T, (c)). Physical photographs of the corresponding graphene aerogels derived from GAH and GAH-T (d).

**Fig. 2:**
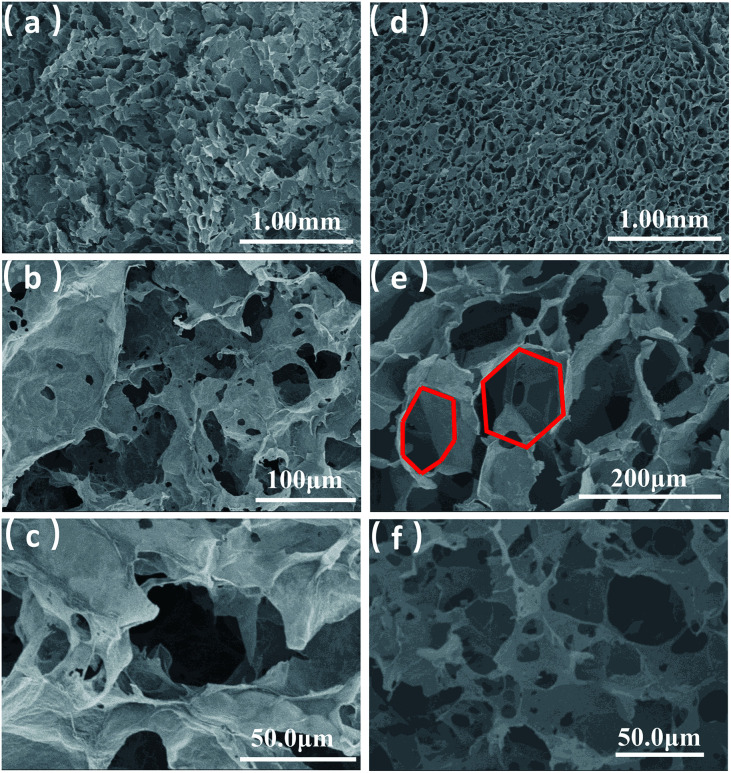
SEM images of GA (a–c) and SEM images of GA-T (d–f).

Under the effect of agitation by a high-speed disperser and with the emulsifier (SDS), the GO suspension was converted to a relatively stable oil-in-water emulsion with cyclohexane and *n*-butanol. The GO edge was rich in different types of oxygen-containing functional groups that made it hydrophilic and its basal plane contained many unoxidized polyaromatic regions that were hydrophobic. Therefore, when the organic materials (cyclohexane and *n*-butanol) and GO sheets were dispersed in water, the organic droplets formed a dispersed phase and the GO sheets adhered to the droplet surfaces (Fig. 1a). An optical micrograph of the aqueous cyclohexane–butanol/GO emulsion is shown in Fig. 1b. Both the height and diameter of GAH-T prepared by the improved soft-template method were larger than those of GAH prepared by the conventional hydrothermal method (Fig. 2c). The graphene hydrogels, GAH and GAH-T, were purged with 20% ethanol and then lyophilized to form graphene aerogels, GA and GA-T, respectively, with their apparent volumes showing almost no change compared to the hydrogels (Fig. 1d).

GA prepared without a soft template exhibited disordered porous structures (Fig. 2a). The graphene sheets were clearly stacked and the pores were irregular and disordered. The pore size distribution was very broad, ranging from a few to 100 microns. The holes were also irregular (Fig. 2b and c), generally with narrow triangle or parallelogram shapes. In contrast, the graphene aerogel prepared using the improved soft-template method (GA-T) exhibited a very uniform pore structure and the pore walls were composed of thin and pleated rGO sheets (Fig. 2d). There was a relatively tight connection between the rGO sheets instead of simple physical stacking, showing their good mechanical strength on a macroscopic level. After the addition of the soft template, the GA-T microstructure underwent a very large change. The rGO sheets were not significantly aggregated and stacked and were divided into individual spherical film forms, which could be generally characterized as a loose porous foam structure (Fig. 2e). Moreover, the size of the holes was uniform and their shape was mostly spherical. Also, the aperture ratio decreased, from 30 to ∼10 μm (Fig. 2f).

### 3.2 Chemical composition of graphene hydrogels and aerogels

**Fig. 3:**
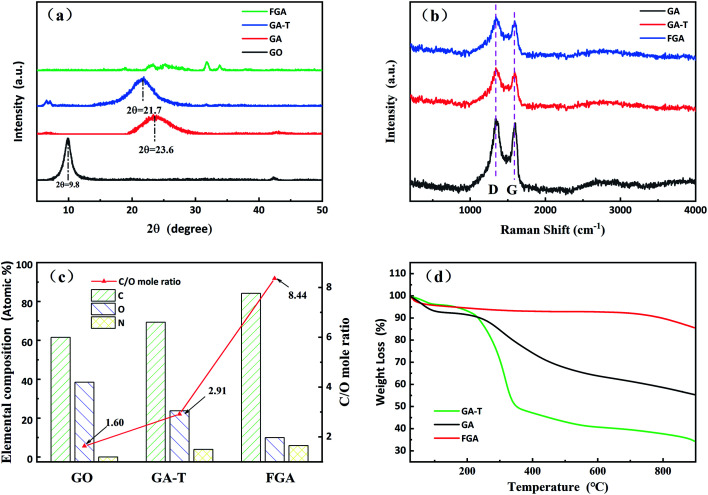
XRD patterns (a) and Raman spectra of the samples (b). Elemental analysis of GO, GA-T and FGA (referring to XPS analysis in Fig. 4 and 5, (c)). TGA curves of GA, GA-T and FGA (d).

The GO transformation to the final aerogels was analyzed by using XRD, Raman spectroscopy, XPS, and TGA (Fig. 3a–d, respectively).

As shown in the XRD patterns, the typical diffraction peak of GO was near 10° and the interplanar spacing calculated by the Bragg equation was ∼0.90 nm. The diffraction peaks of GA and GA-T were at 23.6 and 21.7°, respectively, and, thus, the interplanar spacings of both GA and GA-T had become smaller, indicating that rGO had been formed. FGA was obtained by calcination of GA-T at high temperature in an inert atmosphere. The XRD patterns of FGA showed almost no clear diffraction peaks, indicating that GO was completely reduced (Fig. 3a). In the Raman spectra, GA, GA-T, and FGA all showed a D peak at 1350 cm^−1^ and a G peak at 1500 cm^−1^, indicating the defect density, symmetry, and regularity of the reduced graphene oxide (rGO). The *I*_D_/*I*_G_ values (ratio of the intensities of peak D to peak G) of GA, GA-T, and FGA were ∼1.00/1.05/1.06, respectively. The results showed that the chemical compositions of GA, GA-T, and FGA were very similar. However, the 3D structures (rGO sheet packing structures) of GA and GA-T were very different, which was attributed to the effects of the soft-template (Fig. 2).

The initial C/O (carbon to oxygen atomic ratio) of GO was 1.60/1. After the hydrothermal reaction, GO was converted to GA-T and C/O rose slightly to 2.91/1, indicating that GO reduction was not complete at the lower temperature (Fig. 3c). After calcination at 600 °C, the C/O of FGA increased to 8.44/1, indicating that most of the oxygen-containing functional groups had been eliminated. Notably, there was no N element in GO, however the N content in GA-T was 3.88% when using EDA as the reducing agent. After high temperature treatment, the N content of FGA increased to 5.85% due to further deoxidation.

The TGA curves of GA and GA-T are very different (Fig. 3d). In the range of room temperature to 250 °C, the mass loss of GA and GA-T was mainly due to dehydration. In the range of 250 to 400 °C, the mass loss was attributed to deoxidation of rGO without complete reduction and the mass loss of GA-T was greater than that of GA, which might have been due to the residual SDS in GA-T. After undergoing high temperature calcination, FGA released almost all the organic and unstable substances and, therefore, its mass loss was very low according to TGA.

**Fig. 4:**
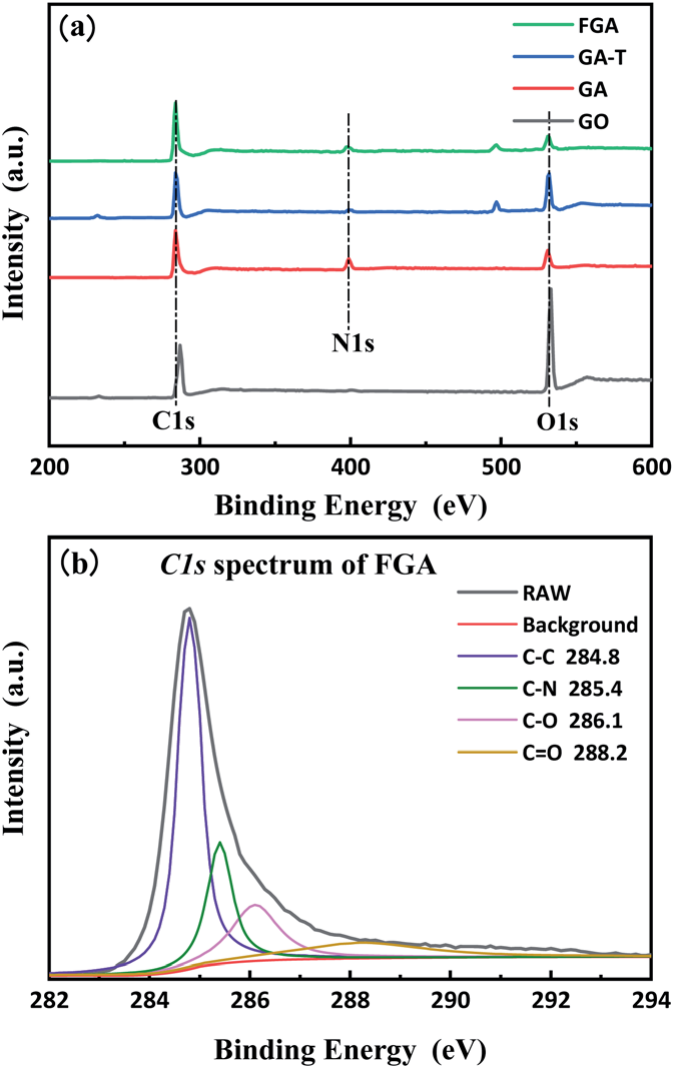
XPS spectra of GO, GA, GA-T and FGA (a) and C-1s XPS spectra of FGA (b).

XPS was used to quantitatively characterize the changes in the composition of the materials during the preparation process as well as microchemical bonding information. The total spectra of GO, GA, GA-T, and FGA are shown in Fig. 4a. The presence of C-1s (285.0 eV) and O-1s (532 eV) peaks appeared in the spectra of all GO, GA, GA-T, and FGA samples. In addition, the spectra of GA, GA-T, and FGA showed N-1s (400.5 eV) peaks, which confirmed that N remained in the aerogels during the reduction of GO with EDA. To determine the relative level of oxidation in the final product FGA, peak fitting of the C-1s spectrum of FGA was carried out using XPS PEAK V4.1 software and four peaks were obtained corresponding to four chemical bonds: sp^2^ carbon (C–C 284.8 eV), carbon–nitrogen (C–N 285.4 eV), epoxy/hydroxyl (C–O 256.1 eV), and carbonyl (C=O 288.2 eV) (Fig. 4b). It was clear that the peak representing carbon bonds was predominant, which indicated that oxygen atoms in FGA had been almost completely removed by calcination at high temperature and the aerogels were highly graphenized. The C–N bond was also found to be the main chemical connection of N atoms in FGA.

**Fig. 5:**
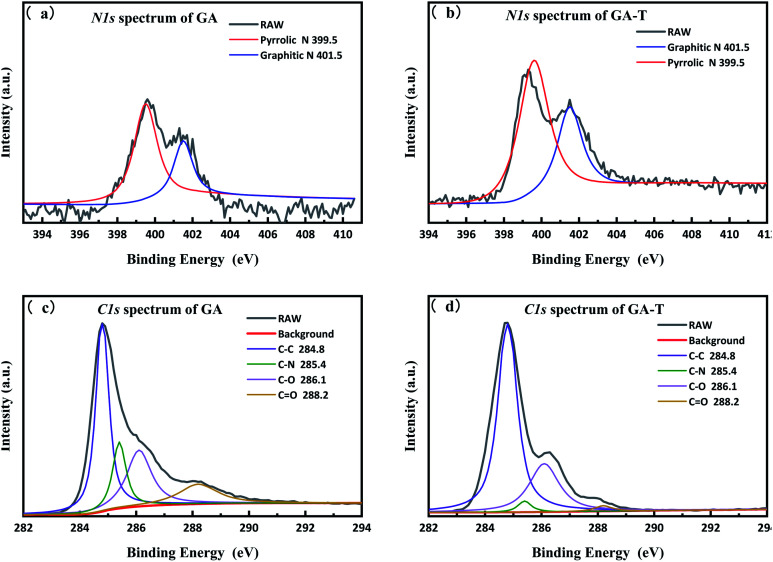
N-1s XPS spectra of GA (a) and GA-T (b) and C-1s XPS spectra of GA (c) and GA-T (d).

To analyse the effects of the soft template on elemental composition, peak fitting of the N-1s and C-1s spectra of GA and GA-T was performed using XPS PEAK V4.1 software, which showed that in both GA and GA-T, the main chemical bonds of N were pyrrole (399.5 eV) and graphitic nitrogen (401.5 eV, Fig. 5a and b). However, the N content in GA (10.6%) was much higher than that in GA-T (3.0%, Fig. S4†). A possible reason for this difference might have been that the dissolving of EDA in the soft template (cyclohexane and butanol) led to less reactions between GO and EDA; in other words, in the soft-template method, the relative concentration of EDA in the GO suspension was decreased and, therefore, the final N content in GA-T was much lower than that in GA. Analysis of the fitted C-1s peaks of GA and GA-T also showed that both GA and GA-T had C–C/C=C, C–N, C–O, and C=O bonds, but the contents of these various chemical bonds were quite different (Fig. 5c and d). The contents of sp^2^ carbon, epoxy/hydroxyl, and carbonyl in GA were 44.9, 25.2, and 13.9%, respectively, while the corresponding contents in GA-T were 68.5, 26.9, and 1.78%, respectively. In comparison, the oxygen content in GA-T was the lowest. With the introduction of the organic solvents as templates, it was believed here that the hydrothermal reaction could be carried out under much greater pressure at the same reaction temperature, thus resulting in a lower oxygen content.

### 3.3 Adsorption capacity of graphene aerogels

**Fig. 6:**
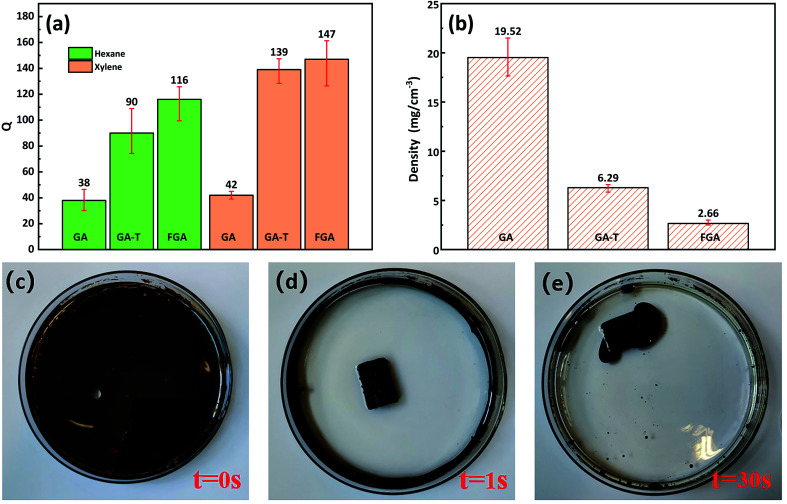
GA and GA-T *Q*s for *n*-hexane and xylene (a). Density of GA, GA-T, and FGA (b). Photo of sewage before adding GA-T (c). Photo of sewage after adding GA-T for 1 s (d). Photo of sewage after adding GA-T for 30 s (e).

In the organic solvent adsorption experiments, the adsorption capacities (*Q*s) of GA-T and FGA for *n*-hexane and xylene were greatly improved compared to that of GA (Fig. 6a). The *Q* of GA-T for *n*-hexane reached 90 times its own weight, which was 136.8% higher than that of GA. For the *Q* for xylene, GA-T reached 139 times its own weight, which was 231% higher than that of GA. After further high temperature calcination, the adsorption capacity of FGA was also improved, but it was not much improved compared to GA. The ability to adsorb organic matter depended on the porosity and density of the organic matter itself. GA-T has a lower density than GA, thus it accommodated more organic matter (Fig. 6b).

The densities of GA, GA-T, and FGA were 19.2, 6.29, and 2.66 mg cm^−3^, respectively, which suggested that GA-T and FGA were ultralight materials (*ρ* < 10 mg cm^−3^, Fig. 6b). GA-T prepared with the soft-template appeared to have a higher porosity and more uniform pore size than GA prepared without template, such that the density of GA-T was much lower. Furthermore, after high-temperature calcination reduction, GA-T lost most of its oxygen atoms and impurities and became FGA, whose density greatly decreased to 2.66 mg cm^−3^.

In the adsorption experiment of simulated spilled oil, the oil was mainly distributed on the water surface and the container wall (Fig. 6c). When GA-T was placed into the contaminated water, the water became clear in 1 s (Fig. 6d) and GA-T adsorbed almost all the oil in the water in 30 s, including oil on the container wall (Fig. 6e). These results demonstrated that GA-T could adsorb oil in the water quickly and efficiently.

## 4. Conclusions

Graphene aerogels were synthesized by a simple one-step hydrothermal method, in which droplets of cyclohexane and *n*-butanol, stabilized with SDS in a GO suspension, were used as soft templates to control the pore shapes and diameters of the final aerogels. Compared with the conventional hydrothermal method, the aerogels prepared in the present study had lower density (∼2.66 mg cm^−3^) and a richer pore structure. In organic adsorption experiments, the newly prepared graphene aerogels absorbed 90 times their weight of *n*-hexane and 139 times their weight of xylene. In the treatment of wastewater containing spilled oil, these graphene aerogels absorbed oil quickly and efficiently. These results show that graphene aerogels prepared with this improved soft-template method possess great potential for applications in wastewater treatment and organic matter separation and recycling from water.

## Conflicts of interest

There are no conflicts to declare.

## Supplementary Material

RA-010-C9RA10988A-s001
