# Supplementary material for: Synthesis of graphene aerogels using cyclohexane and n-butanol as soft templates
Source: RSC Adv. 2020 Apr 8;10(24):14283–90. doi: 10.1039/c9ra10988a (PMC9052014; doi:10.1039/c9ra10988a)
Supplement: RA-010-C9RA10988A-s001 [file RA-010-C9RA10988A-s001.pdf]

Electronic Supplementary Material (ESI) for RSC Advances.
This journal is © The Royal Society of Chemistry 2020

Synthesis of graphene aerogels using cyclohexane and n-butanol as soft template

Xin Zhang [a], Jie Su [a], Xueyuan Wang [a], Xiaolei Tong [a], Fanglian Yao [a,b], and Caideng Yuan*[,a,b]

Supporting

Experimental methods

Synthesis of graphene oxide (GO): Graphene oxide was prepared by a modified Hummers method [1]. Typically, a 9:1 mixture of concentrated H_2SO_4/H_3PO_4(270:30 ml) was added to a beaker with 3 g graphite flakes (80 mesh). The mixture was cooled to 2°C and stirred for 2h, then 13.5g $KMnO_4$ was added to the beaker in batches for about 1h. The reaction was then heated to 50°C and stirred for 6h, after that, 300 ml of water was slowly added to the beaker, producing a severe exotherm to 80-100°C. After the mixture was naturally cooled, the remaining $KmnO_4$ was removed by 10 ml H_2O_2(30%) and produced a lot of bubbles. For workup, the mixture was washed twice with 1 mol/l hydrochloric acid, then was washed with deionized water to neutrality and dried for use.

Characterization of graphene oxide

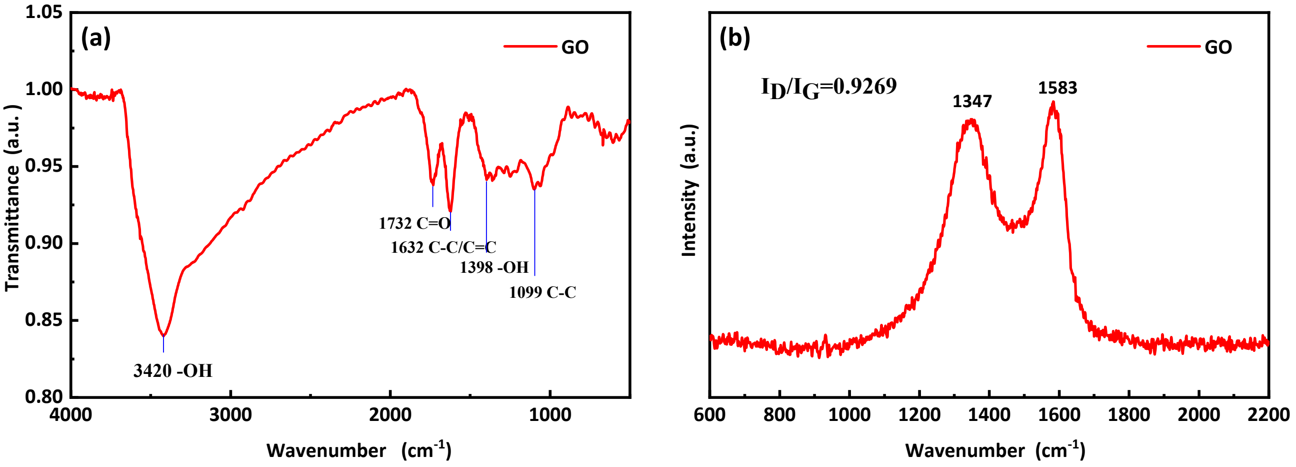

Fig. S1: (a)FTIR spectra of GO, (b) Raman spectra recorded using 514 nm laser excitation

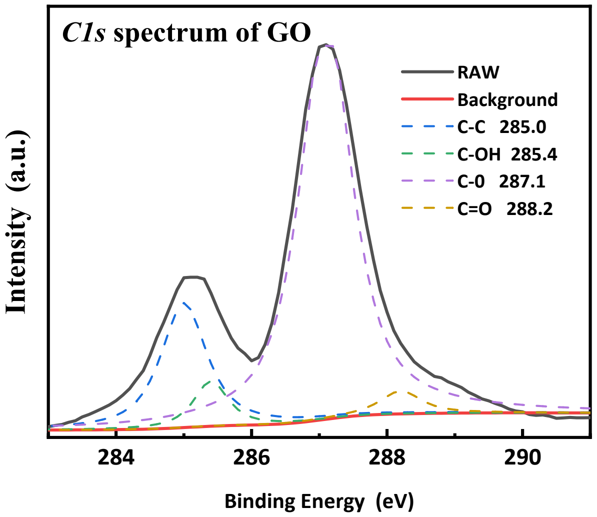

Fig. S2: C1s XPS spectra of GO

[a] Department of Polymer Science and Engineering, School of Chemical Engineering and Technology, Tianjin University, Tianjin 300350, P. R. China.
[b] Tianjin University-UCGM Joint Research Center of Graphene Application, Tianjin University, Tianjin 300350, P. R. China.
* Correspondence author: C. Yuan, e-mail: cdyuan@tju.edu.cn.

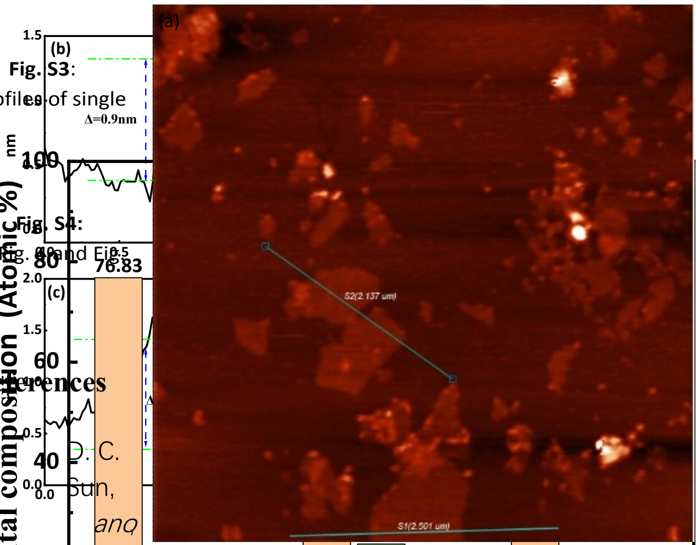

Fig. S3: (a)Tapping mode AFM topographic images, (b) and (c) height profiles of single GO sheets

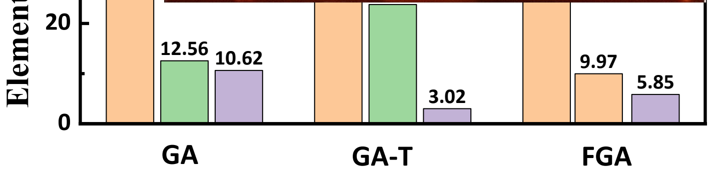

Fig. S4: elemental analysis of GA, GA-T and FGA (referred to XPS analysis of [illegible] 5 in the main document)

References

1. [illegible] D. C. Marcano, D. V. Kosynkin, J. M. Berlin, A. Sinitskii, Z. Z. Sun, A. Slesarev, L. B. Alemany, W. Lu and J. M. Tour, *ACS* [illegible], 2010, **4**, 4806-4814.

This journal is © The Royal Society of Chemistry 20xx

Please do not adjust margins
